# Study of Relationship Between Iron Deficiency and Thyroid Function in Pregnant Females

**DOI:** 10.7759/cureus.32411

**Published:** 2022-12-11

**Authors:** Neha Gupta, Atindra Narayan, Rajinder S Tonk, Shankar K Gupta, Auditi Narayan

**Affiliations:** 1 Department of Medicine, Dr. Baba Saheb Ambedkar Hospital, New Delhi, IND; 2 Department of Medicine, Atal Bihari Vajpayee Institute of Medical Sciences and Dr. Ram Manohar Lohia Hospital, New Delhi, IND; 3 Department of Surgery, Indian Spinal Injury Center, New Delhi, IND; 4 Department of Obstetrics and Gynecology, Faculty of Medicine and Health Sciences, Shree Guru Gobind Singh Tricentenary (SGT) University, Haryana, IND

**Keywords:** thyroid autoimmunity, pregnancy, subclinical hypothyroidism, anemia, thyroid, iron-deficiency

## Abstract

Background: Iron is essential for the normal functioning of thyroid peroxidase and iron deficiency is very commonly encountered during pregnancy. Thyroid disorders and iron deficiency are associated with obstetrical and fetal complications. The aim of the study was to find out the relationship between iron deficiency and thyroid function in pregnant females during first trimester.

Methodology: The present cross-sectional observational study was conducted among first trimester pregnant females at the Department of Medicine, Obstetrics and Gynecology, and Biochemistry at Atal Bihari Vajpayee Institute of Medical Sciences and Dr. Ram Manohar Lohia Hospital, New Delhi. Hundred pregnant women were included in this study. On the basis of serum ferritin value, the study population was divided into two groups namely iron deficient and non-iron deficient. Serum thyroid-stimulating hormone (TSH), FT4, and anti-thyroid peroxidase (TPO) values were then compared between the two groups.

Results: In the present study, 68% of the females were iron deficient. In the Iron deficient group, serum TSH and anti-TPO levels were significantly higher as compared to that in the non-iron deficient group (35.29% vs. 6.25% and 22.06% vs. 3.13%; p=0.001 and 0.018, respectively). A positive correlation was obtained between FT4 and ferritin with correlation coefficient of 0.907 and p-value of 0.0001. Serum TSH levels and serum anti-TPO levels were inversely correlated with ferritin. Univariate logistic regression analysis revealed that iron deficiency was associated with an increased risk of subclinical hypothyroidism with odds ratio (OR) 8.182 (95% CI: 1.798-37.234, p=0.007) and raised anti-TPO with OR 8.77 (95% CI: 1.105-69.681; p=0.040).

Conclusions: Iron deficiency is frequent during the first trimester of pregnancy and is associated with an increased risk of subclinical hypothyroidism and thyroid autoimmunity.

## Introduction

Iron deficiency continues to be a major public health problem in developing countries. According to National Family Health Survey, around 52.2% of pregnant females in India are anemic. It has adverse effects on growth and development, especially in vulnerable groups such as pregnant women and children [1]. Iron deficiency results in the depletion of iron-dependent intracellular enzymes, which participate in many metabolic pathways [2,3]. Iron is a component of thyroid peroxidase (TPO) enzyme, a membrane-bound glycosylated hemoprotein that plays a key role in the biosynthesis of thyroid hormones. This enzyme is also responsible for the oxidation of iodide and the binding of iodine to the tyrosyl residue of thyroglobulin (organification). Iron deficiency may significantly lower the circulating levels of both thyroxine and triiodothyronine, and it may also reduce the conversion of T4 to T3 [3-5]. It has also been reported that iron deficiency increases in vitro hepatic reverse T3 (rT3) deiodination, suggesting increased thyroid hormone metabolism via a deactivating pathway in iron deficiency [6].

Several heterogeneous studies have examined the relationship between iron deficiency and thyroid function in different study groups, such as children, adolescents, and pregnant females [5]. The study population of the present research is comprised of pregnant females, as thyroid disorders and iron deficiency have been found to be associated with many obstetrical and fetal complications [5]. Moreover, very little research has focused on pregnant females to till date, both in India and globally. Additionally, to address the research gap, the present study also explored the effect of iron deficiency on thyroid autoimmunity.

## Materials and methods

The cross-sectional study was conducted at the Department of Medicine, Obstetrics and Gynecology, and Biochemistry at Atal Bihari Vajpayee Institute of Medical Sciences and Dr. Ram Manohar Lohia Hospital, New Delhi. The institutional review board and ethical committee evaluated and approved the study protocol. Informed consent was obtained from all individuals participating in the study.

First trimester pregnant females attending the antenatal clinic were included in the study, whereas second and third trimester pregnant females, females with a known history of thyroid disorder, dimorphic anemia, any ongoing acute infections, or taking iron supplements were excluded.

A total of 113 first trimester pregnant women were screened at the antenatal clinic using convenience and consecutive sampling. Detailed history and physical examination were done. The diagnosis of underlying thyroid disease was evaluated based on the personal history of goiter, thyroid diseases, and/or prior use of thyroid medication. Four women were excluded from the study as they were previously diagnosed with a thyroid disorder. Another four women taking iron supplements were excluded from the study. Three women with a history of fever were also excluded from the study given acute infection given high total leukocyte count. Two women having low hemoglobin levels with a peripheral smear showing dimorphic anemia with low vitamin B12 and folic acid were also excluded from the study. Finally, 100 pregnant women were included in the study.

The study population was divided into two groups based on serum ferritin values. The first group with serum ferritin levels <15 mcg/L was defined as iron deficient (ID). The second group with serum ferritin levels >15 mcg/L was defined as non-iron deficient (NID). The two groups' serum thyroid-stimulating hormone (TSH), free T4 (FT4), and anti-TPO values were then compared. Mean, median, and interquartile ranges were derived for multiple parameters related to demographic and laboratory characteristics. The regression analysis was done between serum ferritin, TSH, FT4, and anti-TPO to find their correlation.

The following normal reference ranges for first trimester pregnancy were used: serum ferritin >15 mcg/L [7-9]; serum TSH <2.5 mIU/L; serum FT4: 0.83-1.27 ng/dL [10]; and anti-TPO <60 IU/mL [11]. Subclinical hypothyroidism was defined as a serum TSH between 2.5 and 10 mIU/L with a normal FT4 concentration [10]. Overt hypothyroidism (OH) was defined as an elevated TSH (>2.5 mIU/L) in conjunction with a decreased FT4 concentration. Women with TSH levels ≥10.0 mIU/L, irrespective of their FT4 levels, were also considered to have OH [10]. Thyroid autoimmunity was defined as serum anti-TPO >60 IU/mL [11].

## Results

A total of 68% (n=100) of the females included in the present study were iron deficient, while 32% (n=100) had normal iron levels. The mean serum ferritin of the females was 17.42±14.02 (3.2-55.4). All the females in the study group had normal free T4 levels. The serum TSH of 74% females was normal, while 26% females had increased serum TSH level. The mean serum TSH was 2.59±1.35 (1.2-8.1). A total of 16% females had increased serum anti-TPO levels, while 84% females had normal serum anti-TPO levels (Table 1). The mean value of anti-TPO was 60.47±49.53 (13.8-220.4). Moreover, 26% females with increased TSH (<10) and normal free T4 were considered to have subclinical hypothyroidism.

**Table 1:** Demographic and laboratory characteristics of the study population (n=100). BMI: body mass index; MCV: mean corpuscular volume; FT4: free T4; TSH: thyroid-stimulating hormone; anti-TPO: anti-thyroid peroxidase

Parameters	Mean±SD	Median	Min-max	Interquartile range
Age (years)	22.44±2.42	22	18-30	20.500-24
BMI (kg/m^2^)	21.17±1.83	21.15	18.2-26.8	19.600-22.350
Period of gestation	8.04±1.85	7.86	5.14-11.43	6.500-9.286
Ferritin (µ/L)	17.42±14.02	10.2	3.2-55.4	7.850-29.050
FT4 (ng/dL)	1±0.15	0.92	0.83-1.26	0.870-1.165
TSH (mIU/L)	2.59±1.35	2.02	1.2-8.1	1.900-2.900
Anti-TPO (IU/mL)	60.47±49.53	46.15	13.8-220.4	34.900-54.450
Hemoglobin	10.12±1.68	9.8	7.1-13.1	8.650-11.800
Total leukocyte count	7174±1469.67	7050	4600-10000	5900-8300
MCV	75.65±9.74	74	59-94	68-84.500
Total serum iron	62±35.33	53.5	10-120	38.500-104
Creatinine	0.75±0.2	0.8	0.3-1.2	0.600-0.900
Urea	25.81±6.31	25	15-43	21-31
Total bilirubin	0.67±0.22	0.7	0.2-1.1	0.500-0.800
Fasting blood sugar	88.41±4.94	88.5	80-98	84-93

As listed in Table 2, the mean value of FT4 in the ID group was 0.9±0.05 (0.83-1.1) and in the NID group was 1.2±0.05 (1.1-1.26). While it was observed to be normal in both groups, the range was lower in the ID group. It was found that FT4 and serum ferritin were significantly associated with each other (p<0.0001). The mean value of serum TSH in the ID group was 2.86±1.54 (1.5-8.1), while it was 2.01±0.45 (1.2-3.7) in the NID group, with a p<0.002. The mean value of serum anti-TPO in the ID and NID groups was found to be 72.14±55.98 (13.8-220.4) and 35.67±11.2 (14.7-68), respectively.

**Table 2:** FT4, TSH, and anti-TPO according to serum ferritin levels. FT4: free T4; TSH: thyroid-stimulating hormone; anti-TPO: anti-thyroid peroxidase

Parameters	Ferritin normal	Ferritin deficient	p-Value
	n=32	n=68	<0.0001
FT4 (ng/dL)	1.2±0.05	0.9±0.05	
TSH (mIU/L)	2.86±1.54	2.01±0.45	
Anti-TPO (IU/mL)	72.14±55.98	35.67±11.2	

Subclinical hypothyroidism was observed in 26% (n=100) females. Of these, 24 females (35.29%) belonged to the ID group, while only two females (6.25%) belonged to the NID group. Elevated levels of anti-TPO antibodies were found in 16% females. Of these, 15 females (22.06%) belonged to the ID group, while only one female (3.13%) belonged to the NID group (Table 3).

**Table 3:** Prevalence of subclinical hypothyroidism and raised anti-TPO. Anti-TPO: anti-thyroid peroxidase

Parameters	All patients	Iron deficient (ferritin <15 mcg/L)	Non-iron deficient (ferritin >15 mcg/L)
N	100 (100%)	68 (68%)	32 (32%)
Subclinical hypothyroidism (TSH >2.5 mIU/L)	26 (26%)	24 (35.29%)	2 (6.25%)
Increased anti-TPO	16 (16%)	15 (22.06%)	1 (3.13%)

A total of 24 females (35.29%) had increased serum TSH levels in the ID group, while only two females (6.25%) had increased serum TSH levels in the NID group. It was found that serum ferritin level was significantly associated with serum TSH level (p=0.001).

**Table 4:** Relationship between serum ferritin and serum TSH. TSH: thyroid-stimulating hormone

Parameters		Ferritin (µ/L)		Total	p-Value
		≤15	>15		
TSH (mIU/L)	Not increased	44 (64.71%)	30 (93.75%)	74 (74.00%)	0.001
	Increased	24 (35.29%)	2(6.25%)	26 (26.00%)	
Total		68 (100.00%)	32 (100.00%)	100 (100.00%)	

In the ID group, anti-TPO was found to be elevated in 15 females (22.06%), while in the NID group, anti-TPO was elevated in only one female (3.13%). It was found that serum ferritin level was significantly associated with serum anti-TPO level, with a p-value of 0.018 (Table 5).

**Table 5:** Relationship between anti-TPO and serum ferritin. Anti-TPO: anti-thyroid peroxidase

Parameters		Ferritin (µ/L)		Total	p-Value
		Normal	Deficient		
Anti-TPO (IU/mL)	Not increased	31 (96.88%)	53 (77.94%)	84	0.018
	Increased	1 (3.13%)	15 (22.06%)	16	
Total		32 (100%)	68 (100%)	100	

**Figure 1:**
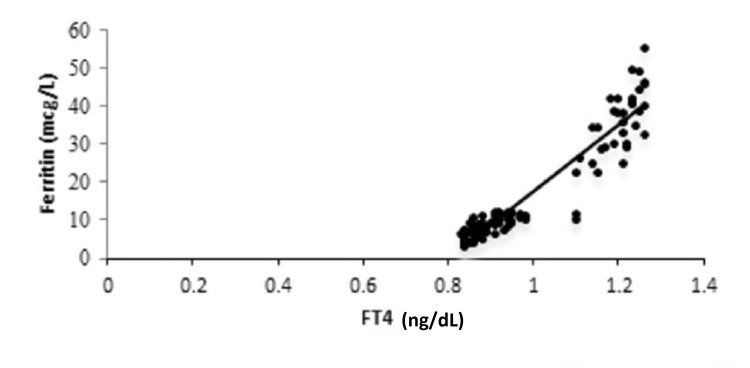
Correlation between ferritin and FT4. FT4: free T4

Among 26 females (n=100) who had subclinical hypothyroidism, 16 females (61.54%) were recorded to have high anti-TPO, while it was not elevated in 10 females (38.46%). Subclinical hypothyroidism and elevated anti-TPO were observed to be significantly associated, with a p<0.0001 (Table 6). A positive correlation was obtained between FT4 and ferritin, with a correlation coefficient of 0.907 and a p-value of 0.0001 (Figure 1).

**Table 6:** Relationship between anti-TPO and subclinical hypothyroidism. Anti-TPO: anti-thyroid peroxidase

Parameters		Subclinical hypothyroidism		Total	p-Value
		No	Yes		
Anti-TPO (IU/mL)	Not increased	74 (100%)	10 (38.46%)	84	<0.0001
	Increased	0%	16 (61.54%)	16	
Total		74 (100%)	26 (100%)	100	

**Figure 2:**
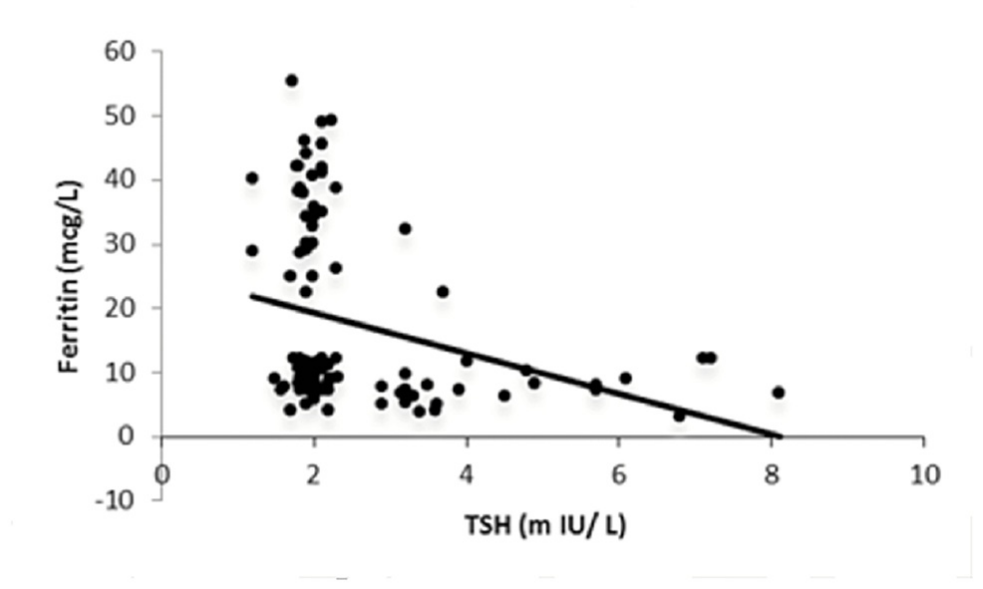
Correlation between ferritin and TSH. TSH: thyroid-stimulating hormone

**Figure 3:**
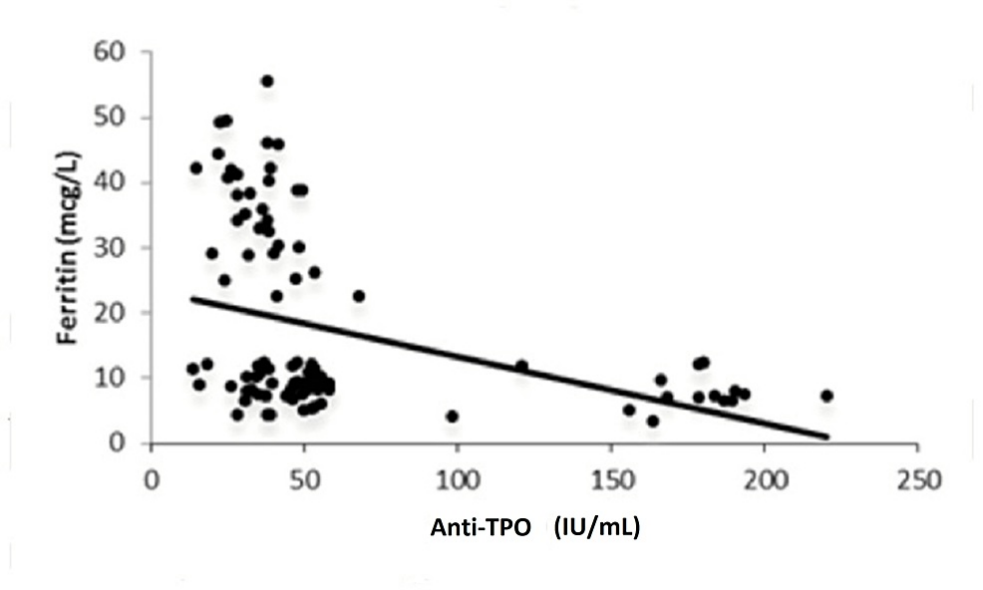
Correlation between ferritin and anti-TPO. Anti-TPO: anti-thyroid peroxidase

It was observed that serum TSH levels and ferritin were negatively correlated, with a correlation coefficient of -0.335 and a significant p-value of 0.0007 (Figure 2). Serum anti-TPO and serum ferritin levels were found to be negatively correlated, with a correlation coefficient of -0.455 and a significant p-value of 0.0001 (Figure 3).

The impact of independent variables ID, age, and BMI on the dependent outcome measures of subclinical hypothyroidism and elevated anti-TPO were explored by fitting logistic regression models. The univariable analysis found that ID was associated with an increased risk for subclinical hypothyroidism, with an odds ratio (OR) of 8.182 (95% CI: 1.798-37.234, p=0.007). No association was noted with age (OR=0.996, 95% CI: 0.827-1.199, p=0.967) or BMI (OR=1.143, 95% CI: 0.897-1.457, p=0.281).

ID was also associated with an increased risk for elevated anti-TPO, with OR=8.77 (95% CI: 1.105-69.681, p= 0.040). No association was noted with age (OR=1.038, 95% CI: 0.834-1.296, p=0.738) or BMI (OR=1.062, 95% CI: 0.795-1.417, p=0.685).

## Discussion

Iron deficiency is the most common nutritional disorder worldwide as well as in India, and its prevention is a public health goal. Studies by Henrichs et al., Haddow et al., and Pop et al. have observed that thyroid dysfunction during early pregnancy has detrimental effects on the neuropsychological development of the offspring [12-14].

A total of 68% of the pregnant females included in the present study were found to be iron deficient, while 32% had normal iron levels. In a study conducted by Veltri et al., the mean serum ferritin was 20 µ/L, and only 35% of the study population was found to be iron deficient [11]. Similarly, Fu et al. found that only 39.06% of the females studied were iron deficient [15].

In the present study, FT4 was found to be normal in all the females, but it was significantly lower in the ID group when compared to the NID group (0.9±0.05 ng/dL vs. 1.2±0.05 ng/dL; p<0.0001). Serum mean TSH levels were significantly higher in the ID group when compared to the NID group (2.86±1.54 mIU/L vs. 2.01±0.45 mIU/L; p<0.002). Elevated TSH levels were found in 35.29% of the ID group compared to 6.25% of the NID group (p=0.001). Serum mean anti-TPO levels were also found to be significantly higher in the ID group compared to the NID group (72.14±55.98 vs. 35.67±11.2; p<0.0001). Anti-TPO values were high (22.06%) in the ID group as well as in the NID group (3.13%), with a p-value of 0.018.

These findings are comparable with those of Veltri et al., who found that the prevalence of thyroid autoimmunity (elevated anti-TPO) and elevated TSH was significantly higher in the ID group compared to the NID group (10% vs. 6% and 20% vs. 16%; p=0.011 and 0.049, respectively) [11]. Serum TSH levels were significantly higher in the ID group (1.5 mIU/L; range: 0.0-9.6 mIU/L) compared to the NID group (1.3 mIU/L; range: 0.0-30.5; p=0.015), while FT4 levels were lower in the ID group (1.0 ng/dL; range: 0.7-2.2 ng/dL) compared to the NID group (1.1 ng/dL; range: 0.6-3.1 ng/dL; p<0.001). Similar results were observed by Fu et al., who found significantly lower FT4 levels in the ID group (p=0.031) compared to the NID group [15]. The mean TSH value of the ID group was significantly higher compared to that of the NID group (p=0.038). Yu et al. found that the median TSH levels were similar in women with ID and in those without ID [16]. However, a significant difference was observed in the serum FT4 levels, which were significantly lower in women with ID than in those without ID (15.37±1.81 vs. 16.38±3.04 pmol/L; p=0.004). The prevalence of mild (18.60% vs. 7.96%; p=0.001) and severe hypothyroxinemia (9.30% vs. 2.67%; p=0.001) in ID patients were markedly higher than in non-ID patients, which was statistically significant.

The present study observed a positive correlation between FT4 and ferritin, with a correlation coefficient of 0.907 and a p-value of 0.0001. Further, it was found that serum TSH levels and ferritin were inversely correlated, with a correlation coefficient of -0.335 and a significant p-value of 0.0007. Serum anti-TPO and serum ferritin levels were also found to be inversely correlated, with a correlation coefficient of -0.455 and a significant p-value of 0.0001. The present results were comparable with the findings of Veltri et al., where serum TSH levels and ferritin were found to be inversely correlated (Spearman’s ρ= -0.076; p=0.001) and a positive correlation was obtained between FT4 and ferritin (Spearman’s ρ=0.112; p<0.001) [11]. Yu et al. showed that the total body iron concentrations were positively correlated with serum FT4 levels (r=0.126; p=0.001), while the total body iron concentrations were negatively correlated with serum TSH levels (r= -0.105; p=0.001) [16].

The impact of independent variables (ID, age, and BMI) on the dependent outcome measures (subclinical hypothyroidism and elevated anti-TPO) was explored using fitting logistic regression models in the present study. The univariate logistic regression analysis found that iron deficiency was associated with an increased risk of subclinical hypothyroidism, with OR=8.182 (95% CI: 1.798-37.234, p=0.007). No association was obtained with age (OR=0.996, 95% CI: 0.827-1.199, p=0.967) or BMI (OR=1.143, 95% CI: 0.897-1.457, p=0.281). Furthermore, iron deficiency was found to be associated with an increased risk for elevated anti-TPO (OR=8.77, 95% CI: 1.105-69.681, p=0.040). No association was noted with age (OR=1.038, 95% CI: 0.834-1.296, p=0.738) or BMI (OR=1.062, 95% CI: 0.795-1.417, p=0.685). Similar results were obtained in a study by Veltri et al., where iron deficiency was found to be associated with an increased risk for thyroid autoimmunity (OR=1.57, 95% CI: 1.11-2.21, p=0.009) and subclinical hypothyroidism (OR=2.31, 95% CI: 1.59-3.37, p<0.001) [11]. No association was observed with age ≥30 years (OR=1.39, 95% CI: 0.98-1.96, p=0.060) or BMI (OR=0.77, 95% CI: 0.48-1.25, p=0.305). Yu et al. found that ID was an independent risk factor for mild (OR=2.440, 95% CI: 1.324-4.496, p=0.004) and severe (OR=3.278, 95% CI: 1.443-7.446, p=0.005) hypothyroxinemia [16].

Strengths and limitations

Through a detailed analysis, the present study examined the association of serum ferritin with TSH, FT4, and anti-TPO. This study was focused exclusively on pregnant females in their first trimester. One of the limitations of this study is its small sample size and the absence of matched controls. Additionally, convenience and consecutive sampling techniques were used for this study as opposed to random sampling. More comprehensive studies can be considered for future research, with the inclusion of a larger population and matched controls. This would help to establish the association in a more robust manner.

## Conclusions

The prevalence of iron deficiency is very high among pregnant females in India. Low iron levels may affect thyroid function, resulting in higher TSH and lower FT4 concentrations in the first trimester of pregnancy. In the present study, 26% of females were diagnosed with subclinical hypothyroidism, while none presented with overt hypothyroidism. A positive correlation was obtained between ferritin and FT4, while a negative correlation was found between serum TSH and anti-TPO levels. Iron deficiency is also associated with a higher prevalence of thyroid autoimmunity during pregnancy. To conclude, proper counseling and treatment of iron deficiency should be provided to females from the pre-conception stage, and early detection of thyroid dysfunction should be encouraged. Assessment of the iron profile, which includes serum ferritin, should also be included in the first trimester evaluation.
